# Admission to hospital for bronchiolitis in England: trends over five decades, geographical variation and association with perinatal characteristics and subsequent asthma

**DOI:** 10.1136/archdischild-2015-308723

**Published:** 2015-09-04

**Authors:** Christopher A Green, David Yeates, Allie Goldacre, Charles Sande, Roger C Parslow, Philip McShane, Andrew J Pollard, Michael J Goldacre

**Affiliations:** 1 Department of Paediatrics, University of Oxford, and the NIHR Oxford Biomedical Research Centre, UK; 2 Unit of Health-Care Epidemiology, Nuffield Department of Population Health, University of Oxford, Oxford, UK; 3 Division of Epidemiology and Biostatistics, University of Leeds, Leeds, UK

**Keywords:** Epidemiology, General Paediatrics, Infectious Diseases, Intensive Care, Respiratory

## Abstract

**Background:**

Admission of infants to hospital with bronchiolitis consumes considerable healthcare resources each winter. We report an analysis of hospital admissions in England over five decades.

**Methods:**

Data were analysed from the Hospital In-Patient Enquiry (HIPE, 1968–1985), Hospital Episode Statistics (HES, 1989–2011), Oxford Record Linkage Study (ORLS, 1963–2011) and Paediatric Intensive Care Audit Network (PICANet, 2003–2012). Cases were identified using International Classification of Diseases (ICD) codes in discharge records. Bronchiolitis was given a separate code in ICD9 (used in England from 1979). Geographical variation was analysed using Local Authority area boundaries. Maternal and perinatal risk factors associated with bronchiolitis and subsequent admissions for asthma were analysed using record-linkage.

**Results:**

All-England HIPE and HES data recorded 468 138 episodes of admission for bronchiolitis in infants aged <1 year between 1979 and 2011. In 2011 the estimated annual hospital admission rate was 46.1 (95% CI 45.6 to 46.6) per 1000 infants aged <1 year. Between 2004 and 2011 the rates rose by an average of 1.8% per year in the all-England HES data, whereas admission rates to paediatric intensive care changed little (1.3 to 1.6 per 1000 infants aged <1 year). A fivefold geographical variation in hospital admission rates was observed. Young maternal age, low social class, low birth weight and maternal smoking were among factors associated with an increased risk of hospital admission with bronchiolitis.

**Conclusions:**

Hospital admissions for infants with bronchiolitis have increased substantially in recent years. However, cases requiring intensive care have changed little since 2004.

**What is already known**

- Bronchiolitis admissions are a great burden to paediatric hospital resources each winter in industrialised healthcare systems.
- Hospitalisation rates for bronchiolitis rose significantly in the USA and Canada in the 1990s.

**What this study adds**

- The annual average episode-based admission rate for bronchiolitis rose sevenfold between 1979 and 2011.
- Since 2004, the estimated all-England annual average hospital admission rate rose by an average of 1.8% each year.
- Bronchiolitis admission rates to paediatric intensive care did not change, implying that increasing hospital admission rates have not been driven by any change in disease severity.
- Record-linkage shows that maternal and perinatal factors are important risk factors for developing severe bronchiolitis and the need for hospitalisation in infancy.

## Introduction

Care of infants with bronchiolitis places a substantial burden on paediatric hospital resources each winter. Infants aged <1 year with bronchiolitis account for 18% of all paediatric admissions.^1^ Infection by respiratory syncytial virus (RSV) is responsible for almost 80% of cases.^2^ Most cases are not severe and can be managed conservatively at home, but approximately 30% of primary infections involve the lower respiratory tract^3^ and supportive care by hospital admission is warranted if feeding or respiration become compromised. Two-thirds of infants have an RSV infection in the first year of life,^3^ 2%–3% of primary infections will require admission to hospital^4^ ^5^ and 2%–6% of these admissions need management on paediatric intensive care units (PICUs).^1^ ^5–7^ Bronchiolitis early in infancy is associated with a higher likelihood of developing wheeze and asthma later in life, and a causal association with asthma remains the subject of enquiry.^8–10^ Worldwide, RSV disease in children under the age of 5 years accounts for 33.8 million lower respiratory infections and 3.4 million hospitalisations. Access to mechanical ventilation on PICUs has helped maintain a low mortality in industrialised nations (0%–1.5% in otherwise healthy infants with RSV).^11^ Globally, an estimated 66 000–199 000 RSV-related deaths occur annually, almost exclusively in resource-poor areas, with RSV being second only to malaria in all-cause infant mortality between 1 and 12 months of age.^12^ ^13^ Prematurity, bronchopulmonary dysplasia, congenital heart disease, immunodeficiency, cerebral palsy and Down's syndrome are recognised risk factors for severe bronchiolitis,^14–16^ but 50%–80% of emergency admissions occur in otherwise healthy infants born at term.^15^ ^16^

There is evidence of a rise in admission rates for bronchiolitis occurring over time in the USA and Canada during the 1990s.^17^ ^18^ We report an analysis of hospital admissions of infants with bronchiolitis in England over five decades. The main objective was to describe a time-trend analysis of admission to hospitals and to PICUs in England. Additional objectives were to map geographical and socioeconomic variation in hospital admission rates. Record-linked data were used to quantify social, maternal and perinatal factors that confer a risk of admission for bronchiolitis, and to measure the risk of later admission for asthma after bronchiolitis.

## Methods

We used discharge codes in any diagnostic position on hospital admission datasets that are routinely collected in England including the Hospital In-Patient Enquiry (HIPE), Hospital Episode Statistics (HES) and Oxford Record Linkage Study (ORLS). Hospital admissions for bronchiolitis were identified using International Classification of Disease (ICD) codes J21.0, J21.1, J21.8 and J21.9 in the 10th revision of the ICD (ICD10, 1999–2011) and 466.1 in ICD9 (1979–1998). In the early years of the ORLS (1963–1978) and HIPE (1968–1978), covered by ICD7 and ICD8, cases were identified from codes 500 (ICD7 until 1965; bronchitis) and 466 (ICD8, 1965–1978; acute bronchitis and bronchiolitis), and therefore covered acute bronchitis and bronchiolitis without distinguishing them for the period before 1979. The relevant codes used for asthma admissions were 493 (ICD8 and ICD9) and J45-46 (ICD10).

National Health Service (NHS) admission statistics from 1968 to 1985 were collected on a one-in-ten basis for the whole of England in the HIPE and scaled up to represent all admissions. Admissions from 1989, including day case admissions, were recorded in HES data. Linked HES data from 1999 permitted an analysis of successive admissions for individuals within the population. The ORLS is essentially a fully linked regional subset of HES, collected independently of HIPE and HES, without sampling, from 1963 to 1998 and compiled as a subset from national HES since then. ORLS covers the period between 1985 and 1989 when no HIPE or HES data were collected. ORLS also includes a specialist maternal dataset, collected between 1970 and 1989 with linkage to later years, which includes maternal and perinatal data not routinely collected through HES and has linked data between mother and baby. Infants admitted with bronchiolitis were compared, in this maternal dataset, with all other infants in ORLS born between 1970 and 1989, using methods described elsewhere.^19^ Some data items—for example, maternal smoking, breast feeding—were not covered in all years. Univariable analysis followed by multivariable adjustment was done for significant variables using logistic regression and the stepwise forward method. A cohort of children with bronchiolitis in HES was compared with a cohort of children without a record of bronchiolitis, using analytical methods for disease associations described elsewhere,^20^ to compare the incidence of asthma admissions when aged between 2 and 14 years.

In the trend analyses, hospital admission rates are presented as episode-based and person-based admission rates of cases per 1000 population, for both sexes combined, aged under <1 year, expressed as an annual rate. The population denominator for each year and each local area was obtained from mid-year population estimates from the Office for National Statistics (ONS). With the episode-based admission rate, a person admitted several times for the condition is counted as many times as he/she has admissions. The person-based admission rate counts individuals once, and only once, at the first admission per year in which they were admitted. In the geographical analyses, confined to 1999–2011 when linked person-based data from HES were available, place of residence was determined from the HES record. The boundaries of the Local Government Areas of England were used to map geographical variations in the recorded rates of admissions and the Index of Multiple Deprivation (IMD 2004) score was used as a measure of socioeconomic status for each local area.

The Paediatric Intensive Care Audit Network (PICANet) dataset is a national audit that has collected data on admissions to all PICUs across England since 2002. We used the primary diagnosis for admission and Read codes X100C, XSDOK, H061, H0615, H061z, X100D, Xa0BK, H0612 and XaPOd to identify admissions for bronchiolitis, deaths and length of stay between 2004 and 2012. The PICANet population denominator for each year was estimated from ONS data. Analyses were performed in SAS (trends, mapping, asthma), GraphPad Prism (PICANet) and SPSS (perinatal study).

## Results

### Hospital admission rates for bronchiolitis over time

**Figure 1:**
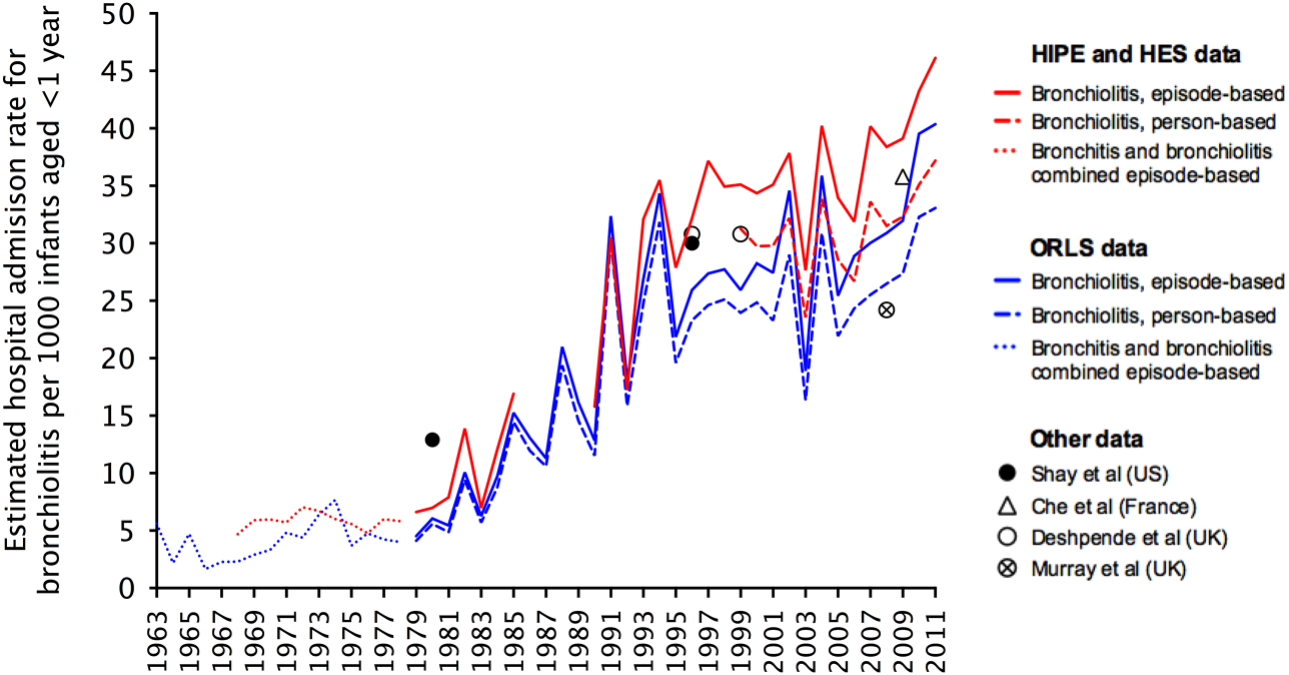
Point estimates for the annual average episode and person-based hospital admission rate for bronchiolitis per 1000 infants aged <12 months from 1979 to 2011, episode-based combined acute bronchitis and bronchiolitis rates from 1965 to 1979 and bronchitis before 1965. Person-based hospital admission rates for males were consistently higher than females (see supplementary material online). 128×63 mm (300×300 DPI). HES, Hospital Episode Statistics; HIPE, Hospital In-Patient Enquiry; ORLS, Oxford Record Linkage Study.

All-England HIPE and HES data recorded 468 138 episodes of admissions for bronchiolitis in infants aged <1 year between 1979 and 2011, accounting for 92.6% of all bronchiolitis admissions in all ages. HES data between 1999 and 2011 recorded 299 397 episodes and 259 044 infants aged <1 year admitted for bronchiolitis. The mean ratio of episodes to people was 1.16. The annual average episode-based admission rate reached 46.1 (95% CI 45.6 to −46.6) in 2011, from 6.6 (95% CI 6.0 to 7.2) in 1979 when bronchiolitis discharge codes came into use (a near sevenfold rise in 32 years) (figure 1). The ORLS recorded a total of 25 852 episodes and 23 319 infants aged <1 year admitted for bronchiolitis in this subpopulation of England between 1979 and 2011. The mean ratio of bronchiolitis episodes to number of infants was lower at 1.11, and 98.2% of all bronchiolitis admissions were in children aged <1 year. Per 1000 infants aged <1 year, the annual average episode-based admission rate reached 40.4 (95% CI 38.5 to 42.3) in 2011, up from 4.5 (95% CI 3.7 to 5.3) in 1979 (a near ninefold rise in 32 years). The trend could be observed before 1979, from when discharge records began 50 years ago. Between 2004 and 2011, when PICU admissions were recorded, the HES and ORLS datasets recorded an episode-based average annual increment in hospitalisation rates of 1.8% and 1.6% respectively. A small, relatively fixed difference between the episode-based and person-based rates of admission emerged from 1995.

### Bronchiolitis admissions to PICU over time

**Figure 2:**
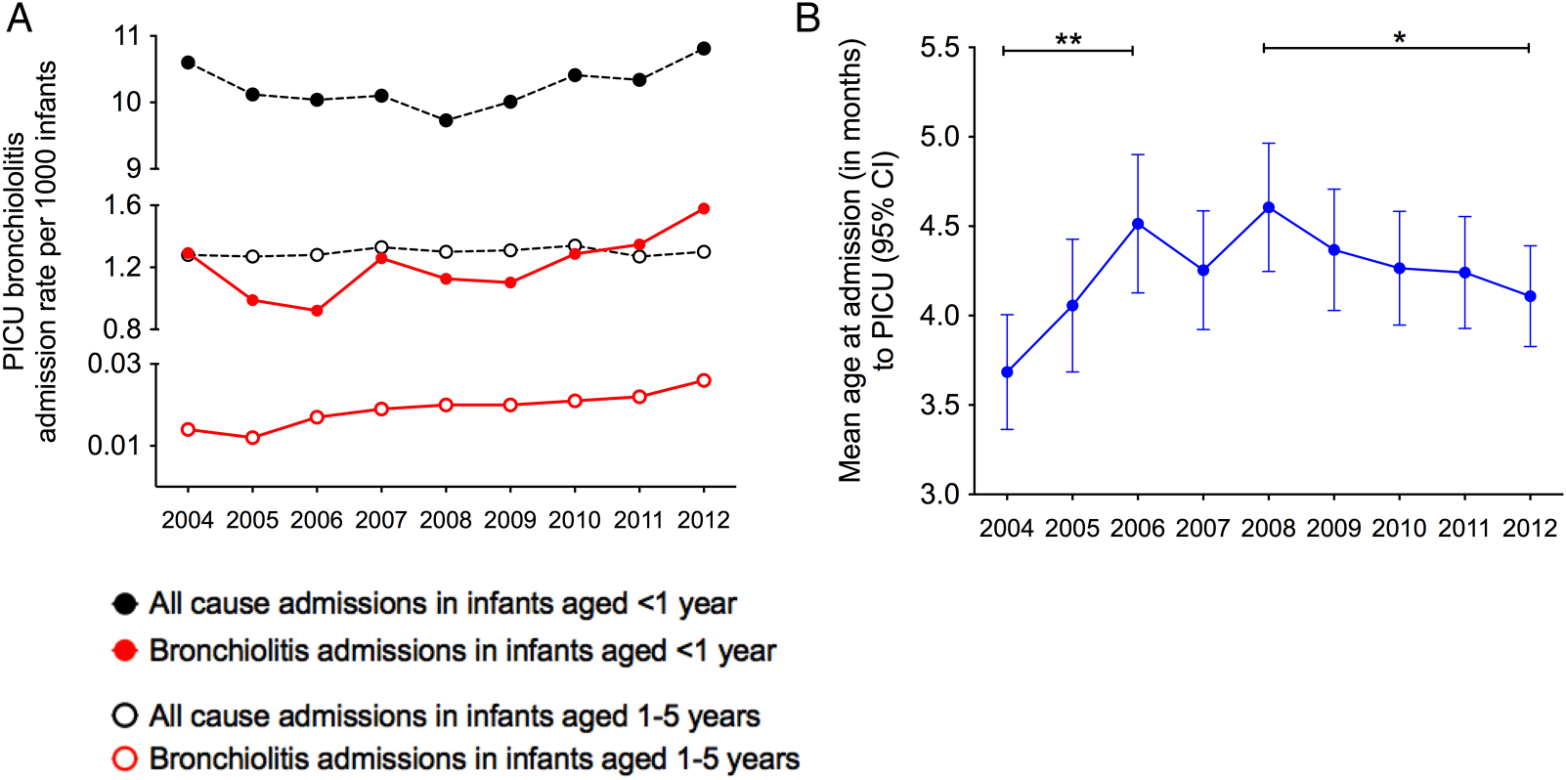
PICU admissions with bronchiolitis and all-causes from 2004 to 2012. (A) All-cause and bronchiolitis admission rates per 1000 infants. (B) Mean age (months with 95% CI) at admission for bronchiolitis. Between 2004 and 2006 the mean age rose significantly (**p=0.0004, two-tailed Mann–Whitney). In more recent years the mean age has fallen significantly between 2008 and 2012 (*p=0.04, two-tailed Mann–Whitney). 124×62 mm (300×300 DPI). PICU, paediatric intensive care unit.

A total of 102 126 infants aged 0–5 years were admitted to PICUs between 2004 and 2012. Of the 64 066 infants aged <1 year, 8172 were diagnosed with bronchiolitis. Infants aged <1 year accounted for 93% of all bronchiolitis admissions and 11.8% (95% CI 10.5% to 13.1%) of admissions each year. The estimated PICU admission rate ranged between 1.3 and 1.6 per 1000 infants aged <1 year with no consistent trend towards increasing admissions (figure 2). The most frequent age at PICU admission was 1 month and 5083/8172 (62.2%) of infants were aged 0–2 months. The annual average length of stay for all ages admitted to PICU with bronchiolitis ranged from 5.4 to 6.7 days (mean 6.1 days). Of the bronchiolitis admissions, 158 resulted in death, representing a PICU case-fatality of 1.75% in infants aged <12 months and 4.4% in infants aged 12 months and older.

### Variation in hospital admission rates for bronchiolitis across England

**Figure 3:**
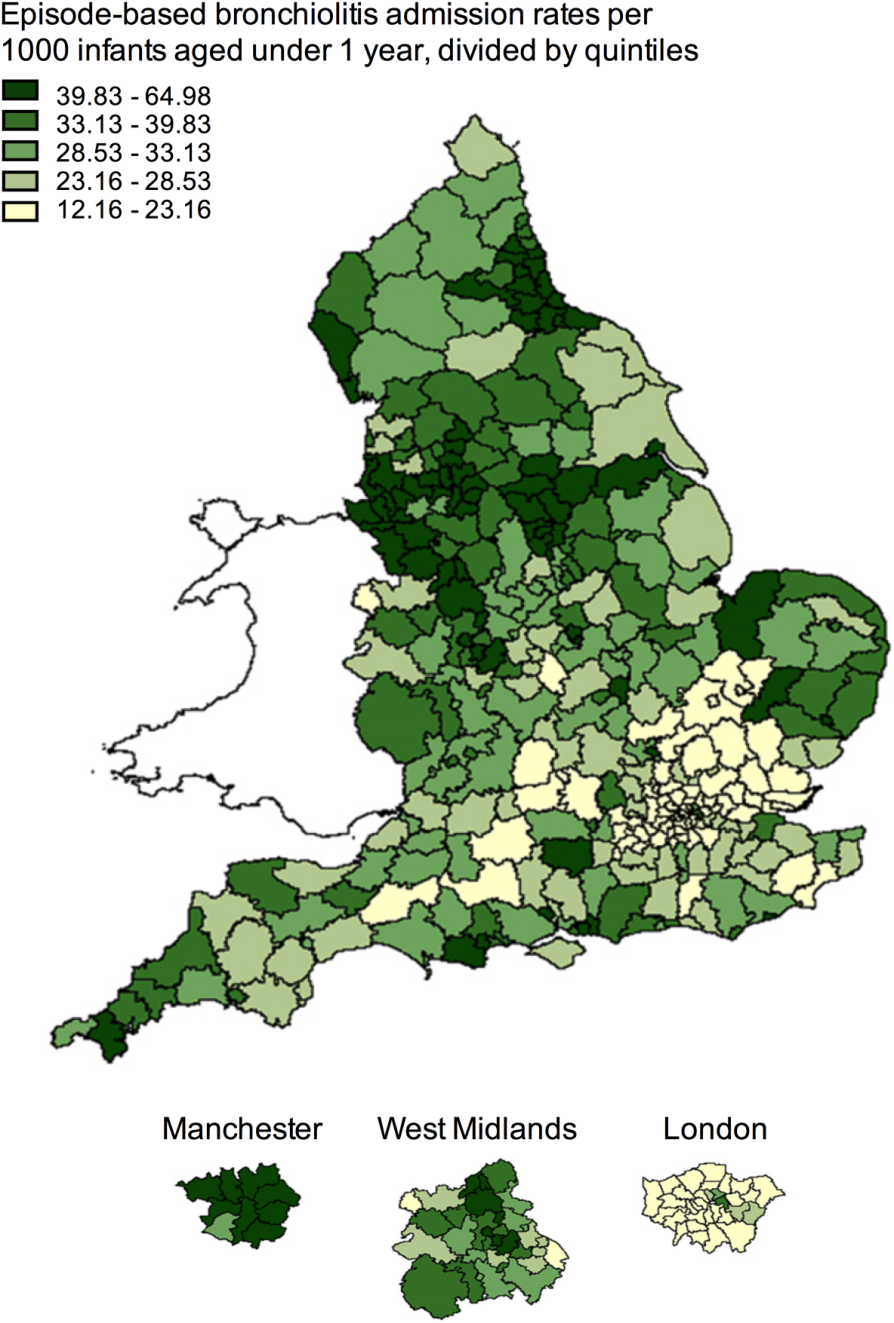
National variation in person-based bronchiolitis hospital admission rates, 1999–2011. Bronchiolitis admission rates per 1000 infants aged <1 year, as analysed using Hospital Episode Statistics, both sexes, mapped using the boundaries of each Local Government Area. 135×173 mm (72×72 DPI).

The mean person-based rate of admissions between 1999 and 2011 was 32.4 per 1000 infants aged <1 year (95% CI 32.2 to 32.5) and ranged 5.3-fold across the 352 Local Government Areas (figure 3). A positive correlation with higher admission rates for bronchiolitis in areas with higher socioeconomic deprivation was observed (see online supplementary material).

### Maternal and perinatal factors associated with hospital admission for bronchiolitis

Of a total of 243 708 infants, 2078 (0.86%) were admitted to hospital with a diagnosis of bronchiolitis. Of these admissions, 88.7% (1852/2087) were aged <1 year (tables 1 and 2). After adjustment of each factor for other significant variables, there were significant associations with year of birth (corroborating the rise in bronchiolitis admission rates over time), low maternal age, low social class, maternal smoking, high parity, twin birth, low birth weight, short gestational age, low birth weight for gestational age, not being breast fed, breech delivery and low Apgar 1 at birth.

**Table 1:** Characteristics of the mother and pregnancy associated with the risk of hospital admission for bronchiolitis in infancy

	Number of children with bronchiolitis (total number with each characteristic)	% with bronchiolitis	p Value	Adjustment for year of birth, social class, mother’s age, parity, smoking and sex: OR (95% CI)
Year of birth				
1970–1974	397 (69 372)	0.57	<0.001	
1975–1979	273 (56 768)	0.48		
1980–1984	530 (60 446)	0.88		
1985–1989	887 (57 122)	1.55		
Total	2087 (243 708)	0.86		
Mother's age (years)				
14–19	260 (17 611)	1.48	<0.001	2.36 (1.81 to 3.08)
20–24	729 (67 447)	1.08		1.42 (1.22 to 1.66)
25–29	648 (90 467)	0.72		1 (1)
30–34	325 (49 544)	0.66		0.87 (0.73 to 1.03)
35–39	106 (15 423)	0.69		0.63 (0.48 to 0.84)
40+	18 (2896)	0.62		0.70 (0.39 to 1.26)
Total	2086 (243 388)	0.86		
Social class				
1 (high)	88 (22 368)	0.39	<0.001	1 (1)
2	289 (46 092)	0.63		1.49 (1.12 to 1.99)
3	723 (87 431)	0.83		1.81 (1.38 to 2.38)
4	286 (25 999)	1.10		2.20 (1.64 to 2.97)
5 (low)	152 (9776)	1.55		2.34 (1.65 to 3.30)
Total	1538 (191 666)	0.80		
Married				
Yes	1675 (220 269)	0.76	<0.001	1 (1)
No	411 (22 996)	1.79		1.13 (0.92 to 1.38)
Total	2086 (243 265)	0.86		
Cigarettes smoked per day				
None	822 (103 596)	0.79	<0.001	1 (1)
1–4	42 (3765)	1.12		1.23 (0.84 to 1.80)
5–14	250 (13 668)	1.83		1.89 (1.59 to 2.25)
15+	298 (12 991)	2.29		2.09 (1.77 to 2.48)
Total	1412 (134 020)	1.05		
Parity				
1	806 (87 340)	0.92		1.93 (1.65 to 2.25)
2	387 (35 342)	1.10		2.26 (1.87 to 2.73)
3+	256 (18 387)	1.39		2.73 (2.17 to 3.43)
Total	2083 (243 159)	0.86		
Pre-eclampsia				
No	1917 (219 636)	0.70	0.006	1 (1)
Yes	168 (24 023)	0.87		1.20 (0.96 to 1.50)
Total	2085 (243 659)	0.86		

Data from the Oxford Record Linkage Study specialist maternity dataset.

**Table 2:** Characteristics of the delivery and infant associated with the risk of hospital admission for bronchiolitis in infancy

	Number children with bronchiolitis (total number with each characteristic)	% with bronchiolitis	p Value	Adjustment for year of birth, social class, mother’s age, parity, smoking and sex: OR (95% CI)
Number of babies				
1	2009 (238 311)	0.84	<0.001	1 (1)
2	76 (5348)	1.42		1.86 (1.37 to 2.54)
Total	2085 (243 659)	0.86		
Birth weight of baby (kg)				
1–1.499	27 (1028)	2.63	<0.001	3.74 (2.32 to 6.01)
1.5–1.999	57 (2745)	2.08		2.34 (1.59 to 3.44)
2–2.499	174 (10 079)	1.73		1.90 (1.50 to 2.41)
2.5–2.999	428 (43 857)	0.98		1.14 (0.96 to 1.35)
3–3.499	777 (95 463)	0.81		1 (1)
3.5–3.999	450 (68 984)	0.65		0.76 (0.64 to 0.89)
4–5.499	163 (20 661)	0.79		0.90 (0.71 to 1.14)
Total	2076 (242 817)	0.85		
Lowest 10th percentile of birth weight for gestational age				
No	1509 (195 332)	0.77	<0.001	1 (1)
Yes	257 (21 694)	1.18		1.34 (1.10 to 1.63)
Total	1766 (217 026)	0.81		
Gestational age (weeks)				
28–36	184 (11 997)	1.53	<0.001	2.24 (1.78 to 2.82)
37	114 (10 275)	1.11		1.62 (1.23 to 2.14)
38	254 (27 314)	0.93		1.23 (0.99 to 1.52)
39	352 (45 299)	0.78		0.97 (0.80 to 1.19)
40	429 (59 596)	0.72		1 (1)
41	246 (42 113)	0.58		0.82 (0.66 to 1.02)
42	105 (12 811)	0.82		1.04 (0.76 to 1.40)
43	32 (3603)	0.89		1.21 (0.76 to 1.95)
44+	57 (4742)	1.20		1.02 (0.67 to 1.55)
Total	1773 (217 750)	0.81		
Infant feeding				
Breast	796 (113 432)	0.70	<0.001	1 (1)
Artificial	758 (49 245)	1.54		1.35 (1.18 to 1.55)
Total	1554 (162 677)	0.96		
Caesarean delivery				
No	1895 (224 350)	0.84	0.026	1 (1)
Yes	183 (18 248)	1.00		1.35 (1.03 to 1.53)
Total	2078 (242 598)	0.86		
Presentation				
Vertex	1510 (153 258)	0.99	0.28	1 (1)
Other	12 (1528)	0.79		1.37 (1.03 to 1.81)
Breech	76 (6578)	1.16		0.76 (0.36 to 1.60)
Total	1598 (161 364)	0.99		
Apgar score at 1 min				
1–7	437 (45 935)	0.95	0.015	1.29 (1.01 to 1.66)
8–9	1288 (146 938)	0.88		1.07 (0.86 to 1.34)
10	209 (28 015)	0.75		1 (1)
Total	1934 (220 888)	0.88		
Apgar score at 5 min				
1–7	34 (2758)	1.23	0.16	1.07 (0.67 to 1.72)
8–9	130 (11 133)	1.17		1.30 (1.05 to 1.62)
10	1360 (134 510)	1.01		1 (1)
Total	1524 (148 401)	1.03		
Sex				
Male	1216 (125 358)	0.97	<0.001	1 (1)
Female	871 (118 344)	0.74		0.74 (0.66 to 0.84)
Total	2087 (243 702)	0.86		

Data from the Oxford Record Linkage Study specialist maternity dataset.

### Hospital admissions for asthma following bronchiolitis in infancy

Nine thousand six hundred and twelve children identified in HES were used as the cohort of admissions with bronchiolitis under the age of 2 years. The control cohort used for comparative analysis numbered 46 377 children. Of these, 715/9612 (7%) children from the bronchiolitis cohort had an admission for asthma before age 14 years. The rate ratio for asthma admissions, comparing the bronchiolitis cohort with the control cohort, was 2.8 (95% CI 2.6 to 3.1).

## Discussion

Data on hospital admission trends for bronchiolitis in the USA, measured per 1000 infants aged <1 year, showed a 2.4-fold rise from 1980 to 1996.^17^ In Canada, between 1981 and 1999, the bronchiolitis admission rate per 1000 infants aged <6 months rose 3.5-fold and 2.1-fold in infants aged 6–12 months.^18^ UK data from a single region between 1996 and 1999 recorded an average rate of 30.8^5^ and the last estimate from an industrialised nation was single-season data from France, which estimated 35.8 admissions per 1000 infants aged <1 year in 2009.^21^ The only all-England estimate recorded an admission rate of 24.2 per 1000 infants aged <1 year^16^ using discharge codes in the primary diagnostic position only, which may explain their lower estimated rate for 2007/08 compared with our data.

We have analysed several datasets over a substantial period and consistently found a substantial rise in hospitalisation rates for bronchiolitis in recent years. In contrast, PICU admission rates have changed little from 2004 to 2012 when hospital admission rates rose by an average 1.8% each year. Although PICU admissions for bronchiolitis have steadily increased as a proportion of all admissions since 2002,^22^ these data suggest that the overall rise in hospital admissions reflects changes in the threshold for admission rather than an increase in disease incidence or severity over time. In the early years of the study, ORLS data collection was independent of that in HIPE and the maternity ORLS dataset was collected separately from the ‘general hospital’ data in the ORLS. These levels of independence increase confidence in the time-trend analyses.

Possible drivers behind changes in recorded hospital admission rates include the increasing prevalence of maternal and perinatal factors that predispose to severe disease, social factors such as parents' expectations, and changes to the way care is provided and used. Improvements in neonatal care have led to an increase in the population at enhanced risk: hospital admission rates are 24.2 for all infants and 47.3 among premature infants per 1000 aged <1 year.^16^ ^23^ ^24^ Even after 36 weeks gestation, our data continue to show an effect of gestational age on the risk of severe disease in single weeks up to weeks 39–41. Before then, each extra week in utero seems to confer added protection against admission with bronchiolitis. In 2003, 7%–9% of bronchiolitis admissions were re-admissions^5^ ^25^ and more recent data estimated that 21% of infants had more than one bronchiolitis admission during the first year of life.^16^ The risk of asthma admission up to the age of 14 years with a preceding history of hospitalisation for bronchiolitis supports previous findings and emphasises the possible wider health consequences of bronchiolitis.^9^

It is noteworthy that overall admission rates in England for children under 15 years of age with lower respiratory tract infections have risen by 40% in the last decade, together with large increases in paediatric admissions for upper respiratory tract infections, urinary infections and gastroenteritis.^26^ These increases were driven by an increase in admissions of short duration (under 24 h). We speculate that major changes in the organisation of healthcare in England may have contributed to changing admission rates. One major change has been decreased access to medical assessment outside hospital as a result of both a reduction in out-of-hours care by general practitioners under a reorganised contract from 2004^27^ and the introduction of a non-medical protocol-based telephone triage (NHS Direct) in 1999.^28^ The potential for these changes to direct parents to hospital care, where availability of new technology over this period, including oximeters,^29^ may have inadvertently encouraged clinicians to lower the threshold for admission to meet waiting time targets and as a result of clinical protocols. We found that only 25% of the geographic variation in hospital admission rates could be attributed to socioeconomic deprivation, which is consistent with two other publications of HES data.^16^ ^30^ The finding of geographical variation and increasing admission rates indicate that there is clinical uncertainty, probably combined with organisational factors, about when to admit a sick child with bronchiolitis.

### Limitations of the study

While the length of the time-period of data collection is a considerable strength of this study, there may have been variation over time in the quality of diagnostic coding and therefore in the accuracy of our data. However, our findings are consistent across different studies both in the UK and elsewhere, suggesting that the large increases in admissions are real. We used PICU admissions as a proxy for severe disease, but other surrogates (such as length of stay in hospital) were not available to infer whether admission rates for cases of mild or moderate disease severity changed over time. Although the overall trend is clear, we noted significant year-to-year variations in hospitalisation rates. Trend data were not available for some factors that may have influenced trends in bronchiolitis rates: these include other conditions that predispose to developing severe bronchiolitis, and demographic factors such as ethnicity.^31^

### Conclusions

Hospital admissions for bronchiolitis in England are increasing and exceed recent estimates from similar industrialised nations. PICU admission rates have changed little over the same period and indicate a need to examine the threshold used for hospital admission and explore the medical and social factors behind the rising admission rates. The high rate of emergency admissions, lack of universal and cost-effective preventative measures and the magnitude of disease incidence make bronchiolitis a major priority for control; these factors also provide a strong rationale for development of strategies for RSV prevention through vaccination.

## Supplementary Material

Web supplement
