# Supplementary material for: Admission to hospital for bronchiolitis in England: trends over five decades, geographical variation and association with perinatal characteristics and subsequent asthma
Source: Arch Dis Child. 2015 Sep 4;101(2):140–6. doi: 10.1136/archdischild-2015-308723 (PMC4752648; doi:10.1136/archdischild-2015-308723)
Supplement: Web supplement [file archdischild-2015-308723-s1.pdf]

FIGURES (SUPPLEMENTARY MATERIAL)

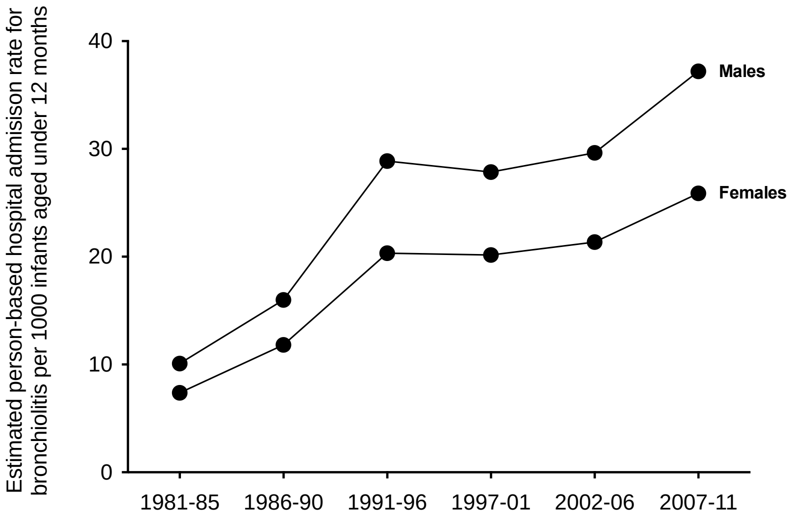

sFigure 1. Male and female person-based bronchiolitis admission rates, infants aged less than 12 months.

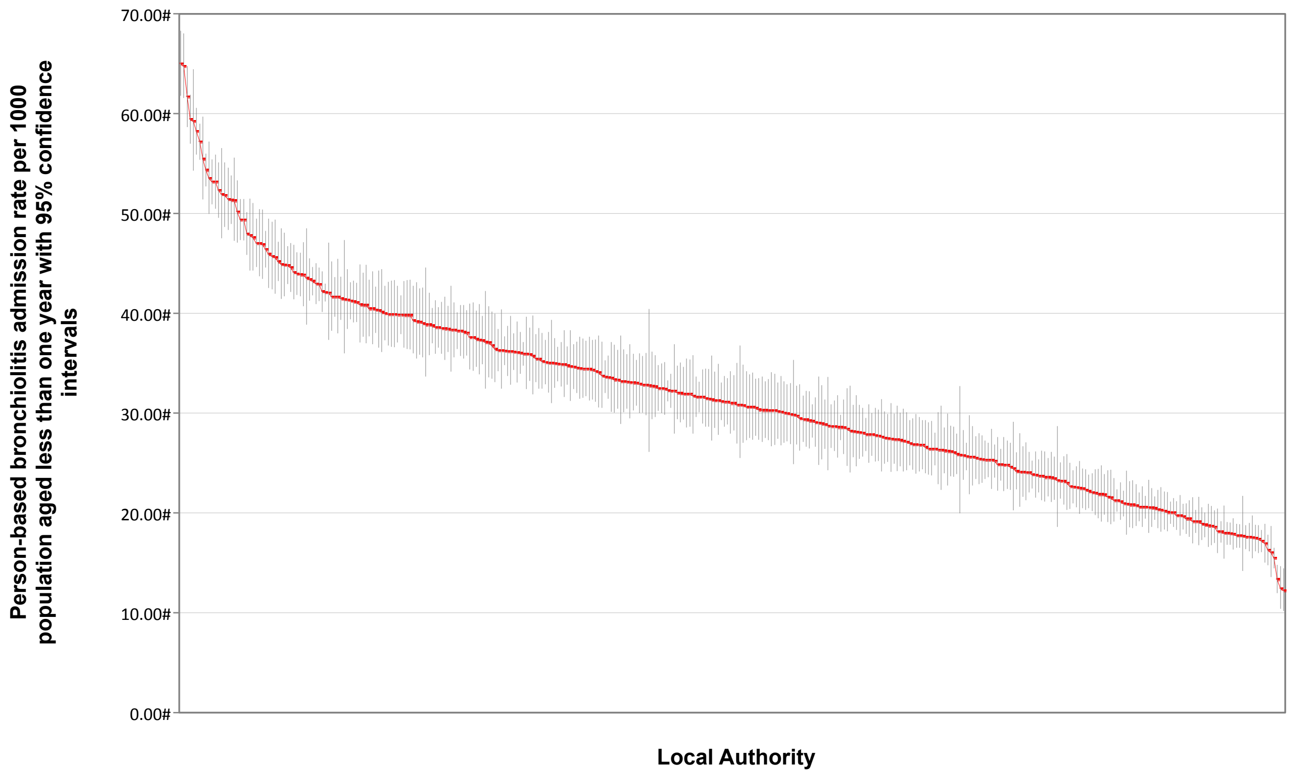

sFigure 2. Person-based bronchiolitis admission rate per 1000 infants aged less than one year for each Local Government Area. 1999 to 2011, males and females combined. Each red dot is the point estimate for a Local Government Area and the vertical bars are the 95% confidence intervals on the point estimates.

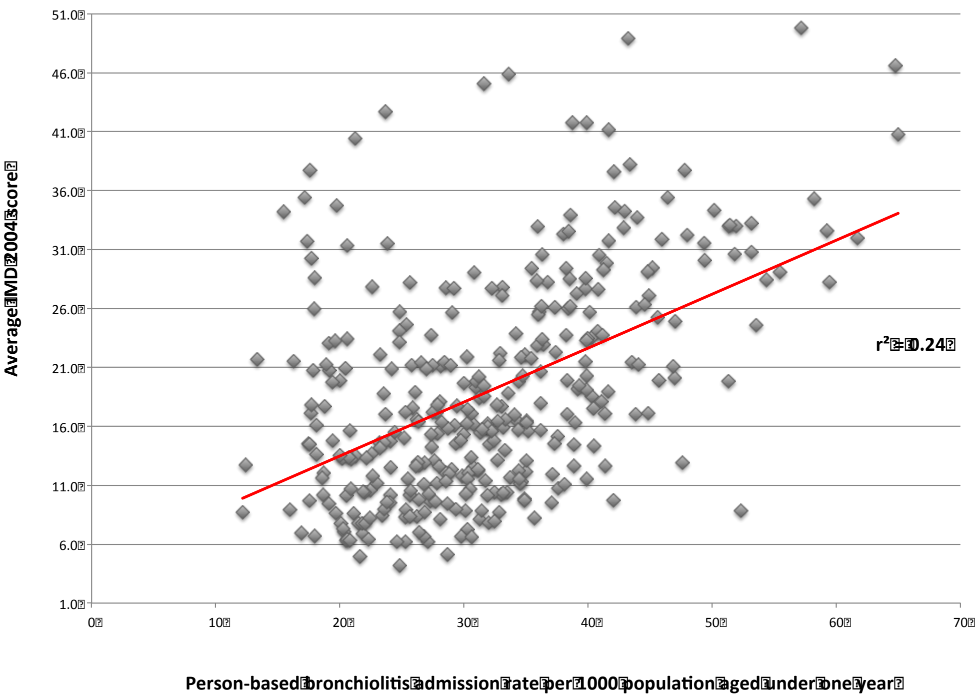

sFigure 3. Index of Multiple Deprivation (IMD) score plotted against the person-based bronchiolitis admission rate per 1000 infants aged less than one year for each Local Government Area. 1999 to 2011, males and females combined.

TABLE (SUPPLEMENTARY MATERIAL)

sTable 1. Annual average episode- and person-based bronchiolitis admission rates per 1000 infants aged less than 12 months from HIPE, HES and ORLS datasets between 1979 and 2011 (95% CI).

There are no all-England data for 1986-89.

Year	Hospital In-Patient Enquiry (HIPE) and Hospital Episode Statistics (HES)		Oxford Record Linkage Study (ORLS)	
	Episode-based rate	Person-based rate	Episode-based rate	Person-based rate
1979	6.61 (5.95 - 7.27)		4.52 (3.72 - 5.32)	4.12 (3.36 - 4.88)
1980	6.98 (6.31 - 7.65)		6.04 (5.14 - 6.95)	5.58 (4.71 - 6.46)
1981	7.88 (7.18 - 8.59)		5.44 (4.58 - 6.31)	4.87 (4.05 - 5.69)
1982	13.87 (12.92 - 14.83)		10.07 (8.87 - 11.27)	9.40 (8.25 - 10.5)
1983	6.96 (6.29 - 7.63)		6.26 (5.32 - 7.21)	5.70 (4.80 - 6.61)
1984	12.08 (11.19 - 12.96)		9.73 (8.56 - 10.9)	8.77 (7.65 - 9.89)
1985	16.90 (15.86 - 17.94)		15.25 (13.8 - 16.70)	14.4 (13.0 - 15.8)
1986			13.07 (11.75 - 14.40)	11.9 (10.6 - 13.2)
1987			11.28 (10.06 - 12.50)	10.5 (9.41 - 11.7)
1988			20.98 (19.36 - 22.61)	19.3 (17.8 - 20.9)
1989			16.15 (14.72 - 17.58)	14.5 (13.2 - 15.9)
1990	15.78 (15.47 - 16.09)		12.83 (11.56 - 14.11)	11.5 (10.3 - 12.7)
1991	30.50 (30.08 - 30.93)		32.36 (30.51 - 34.21)	30.3 (28.5 - 32.1)
1992	17.21 (16.89 - 17.53)		18.10 (16.72 - 19.48)	15.8 (14.5 - 17.1)
1993	32.1 (31.65 - 32.54)		26.88 (25.16 - 28.60)	24.8 (23.1 - 26.4)
1994	35.50 (35.03 - 35.96)		34.34 (32.39 - 36.30)	31.8 (29.9 - 33.7)
1995	27.88 (27.46 - 28.30)		21.84 (20.29 - 23.40)	19.5 (18.1 - 21.0)
1996	32.16 (31.71 - 32.61)		25.93 (24.21 - 27.65)	23.2 (21.6 - 24.9)
1997	37.16 (36.68 - 37.64)		27.37 (25.63 - 29.11)	24.6 (22.9 - 26.2)
1998	34.93 (34.46 - 35.41)		27.73 (25.97 - 29.49)	25.1 (23.4 - 26.7)

1999	35.10 (34.63 - 35.58)	31.27 (30.82 - 31.72)	25.93 (24.22 - 27.63)	23.9 (22.3 - 25.5)
2000	34.35 (33.87 - 34.83)	29.75 (29.30 - 30.19)	28.25 (26.46 - 30.04)	24.8 (23.1 - 26.5)
2001	35.08 (34.59 - 35.58)	29.79 (29.34 - 30.24)	27.43 (25.65 - 29.22)	23.2 (21.6 - 24.9)
2002	37.84 (37.33 - 38.35)	32.19 (31.72 - 32.67)	34.58 (32.55 - 36.61)	29.0 (27.1 - 30.8)
2003	27.64 (27.21 - 28.07)	23.54 (23.14 - 23.94)	18.89 (17.42 - 20.36)	16.3 (14.9 - 17.7)
2004	40.22 (39.71 - 40.73)	33.86 (33.39 - 34.33)	35.88 (33.90 - 37.87)	30.8 (29.0 - 32.7)
2005	33.96 (33.49 - 34.42)	28.59 (28.17 - 29.02)	25.43 (23.77 - 27.10)	21.9 (20.3 - 23.4)
2006	31.88 (31.43 - 32.32)	26.69 (26.28 - 27.10)	28.89 (27.13 - 30.64)	24.3 (22.7 - 25.9)
2007	40.17 (39.68 - 40.66)	33.61 (33.16 - 34.06)	30.02 (28.28 - 31.77)	25.5 (23.9 - 27.1)
2008	38.38 (37.91 - 38.85)	31.52 (31.09 - 31.94)	30.88 (29.15 - 32.61)	26.4 (24.8 - 28.0)
2009	39.10 (38.63 - 39.58)	32.30 (31.86 - 32.73)	31.95 (30.19 - 33.72)	27.3 (25.7 - 28.9)
2010	43.23 (42.73 - 43.72)	35.10 (34.65 - 35.54)	39.52 (37.58 - 41.47)	32.2 (30.5 - 34.0)
2011	46.11 (45.60 - 46.62)	37.18 (36.73 - 37.64)	40.36 (38.42 - 42.31)	33.0 (31.3 - 34.8)
